# Polyanionic Biopolymers for the Delivery of Pt(II) Cationic Antiproliferative Complexes

**DOI:** 10.1155/2016/2380540

**Published:** 2016-09-28

**Authors:** Mauro Ravera, Elisabetta Gabano, Ilaria Zanellato, Elena Perin, Aldo Arrais, Domenico Osella

**Affiliations:** Dipartimento di Scienze e Innovazione Tecnologica, Università del Piemonte Orientale, Viale Michel 11, 15121 Alessandria, Italy

## Abstract

Phenanthriplatin, that is, (*SP*-4-3)-diamminechlorido(phenanthridine)platinum(II) nitrate, an effective antitumor cationic Pt(II) complex, was loaded on negatively charged dextran sulfate (**DS**) as a model vector for drug delivery* via* electrostatic interactions. The free complex and the corresponding conjugate with** DS** were tested on two standard human tumor cell lines, namely, ovarian A2780 and colon HCT 116, and on several malignant pleural mesothelioma cell lines (namely, epithelioid BR95, mixed/biphasic MG06, sarcomatoid MM98, and sarcomatoid cisplatin-resistant MM98R). The* in vitro* results suggest that the conjugate releases the active metabolite phenanthriplatin with a biphasic fashion. In these experimental conditions, the conjugate is slightly less active than free phenanthriplatin; but both exhibited antiproliferative potency higher than the reference metallodrug cisplatin and were able to overcome the acquired cisplatin chemoresistance in MM98R cells.

## 1. Introduction

Cisplatin and its congeners (carboplatin and oxaliplatin) occupy an eminent position in cancer chemotherapy. Unfortunately, their nonspecific targeting and poor delivery cause heavy side effects. For these reasons, optimal delivery of cytotoxic Pt(II) agents is of paramount importance to widen their therapeutic index.

The attachment of drugs to macromolecular carriers significantly alters the physicochemical properties of the resulting conjugates, prolonging their plasma half-life, and, most notably, takes advantage of the Enhanced Permeability and Retention (EPR) effect. This effect, described in the 1980s by Maeda and coworkers, relies on the fact that newly formed tumor vessels are often incompletely organized. The vascular endothelium in tumors proliferates rapidly and has a higher number of fenestrations and open junctions than the normal vessels. Moreover, the lack of a functional lymphatic network prevents efficient removal of excess fluid from solid tumor tissue. The combination of these two effects makes tumors hyperpermeable to the circulating macromolecules, which can extravasate and accumulate in the tumor tissue where they are retained for long periods [1]. The ability to exploit the physiopathologic differences between cancer and normal cells/tissues is at the basis of such a strategy (drug targeting and delivery, DTD).

In the field of Pt-based antitumor drugs, nanosized carriers have emerged in the past few decades as an important way to decrease the side effects of cisplatin and its congeners, maintaining intact their cancer killing ability. In particular, two* N*-(2-hydroxypropyl)methacrylamide (HPMA) copolymer conjugates containing a cisplatin-like “Pt(NH_3_)_2_” or an oxaliplatin-like “Pt(cyclohexane-*1R*,*2R*-diamine)” unit (AP5280 and AP5346 or ProLindac™, resp.) entered clinical development [2]. In this context, several nanoparticles (NPs) carrying Pt(II)-complexes are worthy of mention, since these drug candidates are currently under clinical trials (Aroplatin™ in phase II, Lipoplatin™ in phase II/III, Lipoxal™, and LiPlaCis® in phase I) [3].

Premature detachment of the cytotoxic payload in the bloodstream must be avoided and the drug should be released in tumor cells in response to the microenvironment (e.g., pH or redox potential). In most cases the vector is covalently bound to the drug* via* a carefully designed spacer, but also noncovalent interactions (i.e., hydrogen bonding or hydrophobic or ionic interactions) have been employed [4]. Both approaches offer advantages and disadvantages: covalent linkers need an accurate design of the cleavable arm but the release is generally more tunable; on the contrary, noncovalent interactions are immediately operating, but drugs can be prematurely and randomly released by ion exchange processes in plasma. The size and surface charge of NPs play an important role in determining half-life in circulation and biodistribution [5, 6]. Several examples of ionic conjugates are reported in the literature. For example, doxorubicin, methotrexate, paclitaxel, 5-fluorouracil, and camptothecin have been loaded into micelles via both ionic and hydrophobic interactions [7] or in dendrimers* via* noncovalent entrapment [5].

In seeking charged Pt(II) drugs for testing ionic interactions with suitable complementary carries, one must be aware that, among the platinum(II) amminechlorido derivatives, the uncharged cisplatin is, in principle, the most active antitumor agent, according to the SAR rules, defined by Cleare and Hoeschele [8–10].

Conjugates consisting of polymethylmetacrylate (PMMA) core-shell NPs, bearing cationic (–NH_3_^+^) or anionic (–SO_3_^−^) arms as vectors for [PtCl_3_(NH_3_)]^−^ and [PtCl(NH_3_)_3_]^+^, respectively, were reported by us [11, 12]. In the former case, the* in vivo* antitumor effect of the conjugate was higher than that of cisplatin in inhibiting B16 tumor growth in mice, in spite of the fact that the free anionic drug showed modest antiproliferative activity* per se*, as expected.

**Figure 1:**
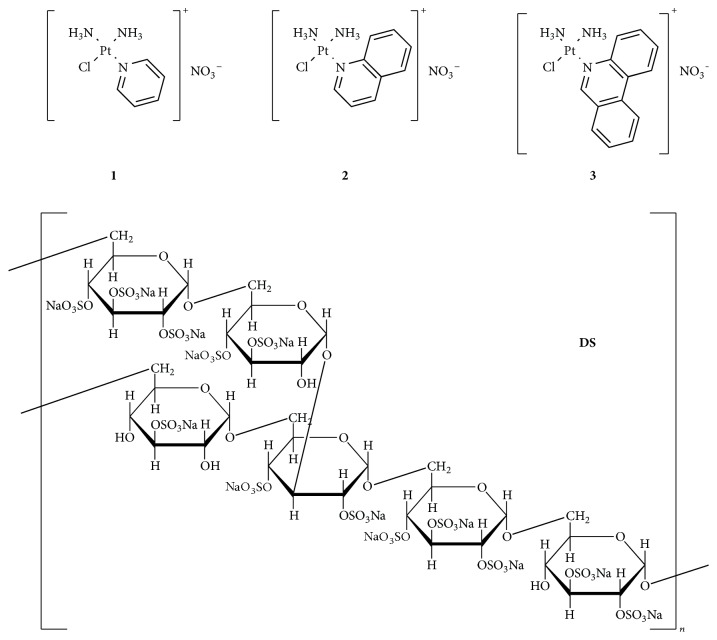
Sketch of the ionic complexes [Pt(Am)Cl(NH_3_)_2_]^+^ (Am = pyridine,** 1**, quinoline,** 2**, and phenanthridine,** 3**) as nitrate derivatives and of dextran sulfate (**DS**).

Recently, Lippard et al. demonstrated that monofunctional platinum(II) complexes of general formula* cis*-[Pt(Am)Cl(NH_3_)_2_]^+^ (Am = pyridine,** 1**, quinoline,** 2**, and phenanthridine,** 3**) (Figure 1) display significant antitumor properties. Their cellular response is different from that of the classic cisplatin-like neutral drugs since they bind to DNA in a monodentate fashion at the N7 position of guanine residues and in the meantime intercalate the DNA helix. The resulting adducts are able to inhibit transcription, while the low distortion of the DNA significantly eludes repair. In particular, phenanthriplatin, that is, (*SP*-4-3)-diamminechlorido(phenanthridine)platinum(II) nitrate,** 3**, exhibited greater activity than the approved drugs cisplatin and oxaliplatin [13] on several tumor cell lines [13–18].

Dextrans are polysaccharides with a linear backbone of *α*(1,6)-linked D-glucopyranosyl residues. The use of dextran as a vector for anticancer drugs has been explored since the '70s and in many cases these conjugates displayed high cellular uptake and good activity, combined with low toxicity [19, 20]. Among charged polysaccharides, dextran sulfate,** DS**, produced naturally by several bacteria or chemically with reaction of dextran with chlorosulfonic acid, is well known for its antiviral and anticoagulant properties. However, concerns about the use of** DS** have been reported, because it may cause anaphylactoid reactions and gastrointestinal complaints [21].

A 500 kDa** DS** has been chosen as a proof of concept that such negatively charged macromolecules are suitable for DTD of cationic Pt complexes** 1**–**3**, since it has been reported that** DS** (especially at high *M*_*r*_) prolonged the circulation time of the corresponding conjugates. Moreover,** DS **conjugates with the anticancer drug doxorubicin (**DOX**) decreased its removal from P-glycoprotein (P-gp), overexpressed in multidrug-resistant cells, and** 3**, having a large heterocyclic ligand, could be the substrate for P-gp driven efflux, in contrast to cisplatin [22].

The ionic interaction between** 1**–**3** and** DS** (Figure 1) is here discussed, with the goal of defining the better experimental conditions for the attaching and the releasing of the cationic drugs to and from the anionic polymer, respectively. The* in vitro* antiproliferative activity of the most promising conjugate, namely,** 3-DS**, has been evaluated on a number of human tumor cell lines. Any EPR effect (relevant for possible DTD strategy) will be obviously appreciable only by means of further* in vivo* experiments.

## 2. Materials and Methods

### 2.1. General Considerations

All chemicals (from Sigma-Aldrich and Alfa Aesar-Johnson Matthey and Co., except where otherwise indicated), including dextran sulfate (**DS**, as sodium salt, from* Leuconostoc *spp.; average *M*_*r*_ > 500,000), were used without further purification. Compounds** 1**–**3** were prepared according to published procedures [13] and their purity was assessed by the usual analytical techniques (elemental analysis, HPLC-MS, multinuclear NMR, etc.). Hyphenated dynamic light scattering (DLS) at a fixed angle (173°) and *ζ* potential experiments were carried out in triplicate on aqueous solutions at 298 K, by using a Zetasizer NanoZS (Malvern, UK) operating in a particle size range from 0.6 nm to 6 *μ*m and equipped by a He-Ne laser (*λ* = 633 nm).

### 2.2. Synthesis of Pt-**DS** Conjugates

The conjugates of complexes** 1**–**3** were prepared by mixing 0.6 mL of an aqueous mother solution of** DS** (10 mg mL^−1^) with different amounts of aqueous solutions of the Pt complexes (from approx. 3 to 30 mL, depending on the desired Pt/S ratio). This procedure was chosen to limit the aggregation of the conjugates. After 15 min stirring, the mixture was dialyzed against water (membrane cut-off = 14 kDa) for 24 h while the external solution was freshly renewed several times. The Pt loading onto** DS** was determined dissolving about 2 mg of the conjugates in 0.8 mL of 70% w/w nitric acid, sonicated for 2 h at 60°C, and diluted with 1.0% w/v HNO_3_ to determine sulfur and platinum content by inductively coupled plasma-optical emission spectroscopy (ICP-OES). A Spectro Genesis ICP-OES spectrometer (Spectro Analytical Instruments, Kleve, Germany) equipped with a crossflow nebulizer was used. In order to quantify the Pt and S concentrations, the 299.797 and 180.731 nm lines were selected. Standard stock solutions of 1000 mg L^−1^ were diluted in 1.0% w/v nitric acid to prepare calibration standards for both elements.

### 2.3. Pt Release from** 3-DS**

The stability of the phenanthriplatin-**DS** conjugates (**3**-**DS**) and the corresponding Pt release were verified at 25°C by dissolving them in 0.5x, 1x, or 2x phosphate buffered saline, PBS (1x PBS = Na_2_HPO_4_ 8.1 mM; KH_2_PO_4_ 1.76 mM; NaCl, 137 mM; KCl 2.7 mM), in a dialysis bag (membrane cut-off = 14 kDa). The Pt released in the external buffer solution was monitored by means of UV-visible spectroscopy (*λ*_max_ for** 3** = 251 nm, *ε* = 34433 ± 87 M^−1^ cm^−1^). UV-visible spectra were recorded with a JASCO V550 spectrophotometer.

### 2.4. Cell Culture and Growth Inhibition (IC_50_)

Cisplatin, free** 3,** and** 3-DS** were tested on the ovarian carcinoma cell line A2780 and the colon carcinoma cell line HCT 116 (European Collection of Cell Cultures, UK). Additionally, the above-said drug candidates were tested also on three primary malignant pleural mesothelioma cancer cell lines, derived from pleural effusion of patients with histologically confirmed mesothelioma (i.e., BR95 epithelioid-, MM98 sarcomatoid-, and MG06 mixed/biphasic-phenotype) and on the cisplatin-resistant cell line MM98R [23–25]. The following media were used to culture the cells: RPMI 1640 (for A2780 cells), McCoy's 5A (for HCT 116 cells), Ham's F10 (GIBCO, Invitrogen Life Science, San Giuliano Milanese, Italy, for BR95 and MG06), and Dulbecco's modified Eagle's medium (for MM98 and MM98R). In all cases, the media were supplemented with L-glutamine (2 mM), penicillin 100 IU mL^−1^, streptomycin (100 mg L^−1^), and 10% fetal bovine serum (FBS). Mother solutions of cisplatin and complex** 3** were obtained by dissolving the compounds in a 0.9% w/v NaCl aqueous solution brought to pH 3 with HCl (final stock concentration 1 mM). The dialyzed solution** 3-DS** was used as a stock solution. The three solutions were diluted in complete medium to the required concentration range and the cells were challenged at 37°C in a 5% CO_2_ humidified chamber with the compounds under study (72 h, continuous treatment). A standard cell viability test (i.e., the resazurin reduction assay) was used to assess the growth inhibition of the compounds [26]. Details of the protocol have been reported elsewhere [27].

### 2.5. Cellular Accumulation

The cellular accumulation experiments were performed according to already published procedures [28]. Briefly, A2780 cells were treated with 10 *μ*M concentrations of the compounds/formulations under investigations in 10 mm Petri dishes or 175 cm^2^ flasks. After 4 h, the cells were washed with PBS, detached from the Petri dishes by using 0.05% Trypsin 1x + 2% EDTA (HyClone, Thermo Fisher), and transferred into a glass tube. Centrifugation and careful removal of the supernatant gave the cellular pellets that were stored at −20°C until mineralization. After defrosting, the cells were treated with 70% HNO_3_ (for 1 h at 60°C in an ultrasonic bath) and the acid was diluted to a final 1% concentration. The Pt content was measured by inductively coupled plasma-mass spectrometry (ICP-MS, Thermo Optek X Series 2). The concentration of Pt found in cells after the treatment was normalized upon the cell number and the cellular volume, in order to obtain the intracellular Pt concentration. The ratio between the intra- and the extracellular (in the culture medium) Pt concentration is defined accumulation ratio, AR [29].

## 3. Results and Discussion

### 3.1. Synthesis of Pt-**DS** Conjugates, Pt Loading, and Pt-**DS** Size

Complexes** 1**–**3** were synthesized according to literature procedures. Briefly, upon reaction between cisplatin and silver nitrate in DMF, the resulting* cis*-[PtCl(NH_3_)_2_NO_3_] intermediate reacted with the corresponding heterocyclic ligand to give the final product. The identity and the purity of the complexes were checked by elemental analysis, HPLC-MS, and multinuclear NMR that were in agreement with previously published data [13].

**Figure 2:**
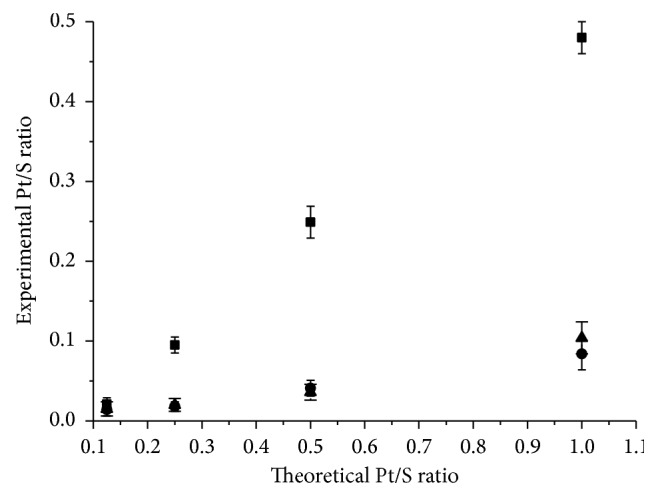
Experimental versus theoretical Pt/S molar ratios for the interaction between** 1** (triangles),** 2** (circles), and** 3** (squares) with** DS**.

The Pt-**DS** conjugates of complexes** 1**–**3** were prepared by adding the solution of each complex to an aqueous solution of commercial 500 kDa** DS**. Importantly, the Pt concentration should not exceed 1 mM in the final solution, in order to prevent quick and massive aggregation. After stirring for 15 min, the solutions were dialyzed for 24 h against water. The resulting solution can be used as it is or eventually lyophilized to obtain a dried powder. Various Pt-**DS** samples were synthesized and characterized according to their different Pt/sulfate (evaluated as Pt/S) ratio by inductively coupled plasma-optical emission spectroscopy (ICP-OES) (Figure 2).

The interaction of** DS** with** 1** and** 2** was quite similar and only a small amount (max. 10%) of the Pt complex was bound to the carrier even when Pt was added in 1 : 1 molar ratio with respect to the sulfate groups present in** DS**. On the contrary, a linear relationship between theoretical and experimental Pt/S ratio is observed for the bulkier complex** 3**, reaching up to the 50% Pt loaded. This is in contrast with the occurrence of ionic interaction only, since the charge density is in the order** 1** >** 2** >** 3**. These features seem to point out that additional forces are involved in the formation of the conjugate. In agreement with what was reported for** DOX**-**DS** conjugates [30], beyond electrostatic interactions, *π*-*π* stacking between drug and** DS**, together with some additional hydrogen bonding, may also occur (see below for further comments). In particular, *π*-*π* stacking can be significantly more marked in the case of** 3** with respect to the other complexes, justifying its higher loading.

Since** 3** shows higher loading than the others and, moreover, it is the most cytotoxic agent of this class of cationic complexes [13], the following study was focused on** 3 **only.

In this context, the conjugate containing the Pt/S final molar ratio of 33 ± 2 %, hereafter named** 3-DS**, showing excellent water solubility, was used throughout (0.76 mg of** 3** per mg of native** DS**).

In order to verify the size of the above-said conjugate, both a freshly dialyzed solution and a sample, obtained after lyophilization and water redissolution, were analyzed by dynamic light scattering (DLS). The lyophilized sample showed a multimodal dimension distribution with the main set having mean diameter of about 1280 nm. Thus, the lyophilization of the sample favored the formation of larger aggregates and, therefore, this procedure was discarded.

On the contrary, the freshly dialyzed solution exhibited particles with mean diameter of about 120 nm (**DS** alone in such experimental conditions showed mean diameter of about 250 nm). The fact that the conjugate** 3-DS** has smaller size than** DS** can be ascribed to remarkable interactions between** 3** and** DS**, which cause a more compact arrangement in the conjugate.

The freshly dialyzed solution containing such conjugate, hereafter named** 3-DS**, was used for the biological tests. Noteworthily, NPs with diameter ranging from 50 to 150 nm are ideally suitable for exploiting EPR effect [31].

DLS measurements, performed in water during 14 days after synthesis (the time interval for total release of** 3** from** 3-DS**; see below), indicated that the nanoparticle size was maintained throughout, testifying the good stability of** 3**-**DS**. Hyphenated *ζ* potential measurements showed that both** DS** and** 3**-**DS** exhibit negative values (in the range −40 to −50 mV) due to the remaining free sulfate groups, thus explaining the stability of the suspension by virtue of interparticle repulsion forces.

### 3.2. Pt Release from** 3-DS**

**Figure 3:**
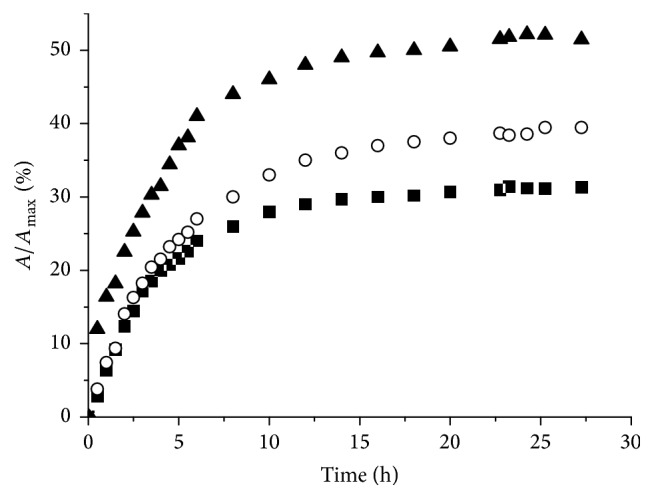
Determination of the Pt released from** 3**-**DS** in 0.5x (black squares), 1x (white circles), and 2x PBS (black triangles) solutions (pH = 7.4), at different times. *A* = absorbance of the solution at different time points (*λ*_max_ = 308 nm); *A*_max_ = theoretical absorbance of the solution considering a complete release of the loaded Pt complex.

The stability in pseudobiological conditions is an important feature for the investigated conjugate. Therefore, the behavior of** 3-DS** was studied at 25°C as a function of time in phosphate buffered saline (PBS) at different ionic strengths, obtained from different dilutions of the mother PBS solution (Figure 3). The concentration of free** 3** in solution was followed by UV-visible spectroscopy: its release was directly correlated with the ionic strength of the medium.

For all PBS concentrations, the corresponding curves reached a sort of plateau after 10–15 h each with different residual loading, corresponding to about 50%, 40%, and 30% of drug release, respectively. Then, the release of** 3** from** 3-DS** smoothly continued and was almost complete within two weeks.

The observed behavior is similar to that reported for** DOX**-**DS **conjugate, where the electrostatic interaction between negatively charged** DS** and positively charged doxorubicin is strongly salt-dependent. In the actual case, increasing the ion strengths of the medium, the cations (Na^+^ and K^+^) and anions (Cl^−^ and HPO_4_^2−^), present in the PBS buffer, compete for the electrostatic interaction with negatively (**DS**) and positively (**3**) charged species, respectively. Additional forces than ionic interactions should be responsible for the slow release of the remaining** 3**, observed on a longer time scale. Likely, these interactions consist of *π*-*π* stacking between adjacent aromatic-structured molecules of** 3**, forced at short distance by their initial amassing on** DS** pivoted by electrostatic interactions. Moreover, hydrogen bonding interactions, involving the N–H groups of ammonia ligands and sulfate groups, may play also an important role, in a fashion similar to that found between ammonia ligands and phosphate groups in cisplatin-DNA adducts [32].

A similar scenario, associated with the presence of multiple interaction forces, was indeed observed for** DOX-DS** [30].

### 3.3. Antiproliferative Activity and Cellular Accumulation

Complex** 3** and its conjugate** 3-DS** (freshly dialyzed) were tested on two cell lines derived from common human tumors (namely, ovarian A2780 and colon HCT 116 carcinomas) and on human malignant pleural mesothelioma (MPM) cell lines (namely, epithelioid BR95, mixed MG06, sarcomatoid MM98, and sarcomatoid cisplatin-resistant MM98R). MPM is a kind of tumor that is very difficult to treat: the current gold-standard frontline chemotherapy approved by the Food and Drug Administration (FDA) consists of cisplatin combined with the antifolate pemetrexed [33]. New drugs and tailored treatments are highly warranted to improve the outcome of MPM patients. The results obtained with the studied compounds are reported in Table 1. As expected, the best results were observed on the cisplatin-sensitive A2780 cells. The good ability to bypass cisplatin resistance (RF) in MM98R cell line of** 3** is maintained by the conjugate** 3**-**DS**. In general, both are more active than reference cisplatin, but free** 3** is slightly more active than** 3**-**DS**. The differences observed for the free and conjugated form of** 3** are related at first instance to the incomplete release of** 3** from** 3**-**DS** conjugate within the time interval of the viability tests. Moreover, the cellular Pt accumulation of the two forms of** 3** must be considered (Table 1). In fact, a key parameter in understanding the mechanism of action of a drug is the evaluation of its intracellular accumulation, which is the final balance between cellular influx and efflux [34]. This parameter is here expressed as accumulation ratio (AR), the ratio between the intracellular Pt concentration and the extracellular (in the culture medium) Pt concentration at a given time [35]. The AR values for** 3**,** 3-DS,** and cisplatin as a reference metallodrug were measured on A2780 cells after 4 h of continuous treatment (Table 1).

The** 3-DS** conjugate shows slightly less intracellular Pt accumulation than the free** 3**, albeit both are internalized more efficiently than cisplatin. As a rule of thumb, positively charged NPs show higher uptake through endocytosis pathways than negatively charged counterparts [36–38] and, unfortunately, the** 3-DS** conjugate has an overall negative *ζ* potential. In contrast, the cationic polymer bearing the anionic [PtCl_3_(NH_3_)]^−^ complex showed remarkable cellular uptake,* via* highly efficient endocytosis of the conjugate due to its residual positive charge [11].

## 4. Conclusions

In this work, the active cationic Pt(II) complexes [Pt(Am)Cl(NH_3_)_2_]^+^ (Am = pyridine,** 1**, quinoline,** 2**, and phenanthridine,** 3**) were allowed to react with an anionic macromolecular carrier, that is, dextran sulfate** DS**, forming conjugates mainly* via* electrostatic interactions.

Complex** 3** showed the higher loading efficiency of the series, and its conjugate** 3-DS** released a substantial part of the active species** 3** in the first 10–15 h depending on the ionic strength of the medium. However, a fraction of** 3** remains tightly bound to** DS** pointing out that different interaction forces, likely *π*-*π* stacking between adjacent aromatic-structured molecules of** 3**, are overlapped to the electrostatic attraction.

Free** 3** and the conjugate** 3-DS** showed in all tumor cell lines IC_50_ values lower (i.e., higher antiproliferative activity) than those of the reference metallodrug cisplatin and, importantly, were able to bypass acquired cisplatin resistance on MM98R cells.

The slight lower* in vitro* antiproliferative activity of conjugate** 3-DS** with respect to free** 3** is due to the incomplete release of** 3** from** 3-DS** within the time interval of the viability tests and to the lower cellular accumulation of** 3-DS**.

Further* in vivo* tests will be necessary in order to appreciate any EPR effect for** 3-DS**.

## Figures and Tables

**Table 1:** Antiproliferative activity (IC_50_ values, *μ*M) after 72 h continuous treatment and cellular accumulation (AR, 4 h, 10 *μ*M) of cisplatin, complex **3**, and its conjugate **3-DS**.

Complex	IC_50_ (*μ*M)						AR (A2780)
	A2780	HCT 116	BR95	MG06	MM98	MM98R	
Cisplatin	0.46 ± 0.11	2.32 ± 0.35	6.2 ± 0.9	4.1 ± 1.5	3.2 ± 1	19.4 ± 2.8 (6.1)	0.62 ± 0.12
**3**	0.12 ± 0.02	0.76 ± 0.09	1.32 ± 0.02	0.72 ± 0.14	0.86 ± 0.12	2.09 ± 0.06 (2.4)	4.81 ± 1.08
**3-DS**	0.20 ± 0.04	1.19 ± 0.02	2.13 ± 0.29	1.03 ± 0.09	1.83 ± 0.11	3.46 ± 0.36 (1.9)	3.03 ± 0.57^*∗*^

Data are means ± SD of at least three independent experiments performed in triplicate. Data in brackets are the resistance factor RF = IC_50_ (MM98R)/IC_50_ (MM98). The AR value of **3-DS** was compared to that of free **3** by means of the two-sample *t*-test (^*∗*^ *p* < 0.5).
